# Effect of astragalus injection on renal tubular epithelial transdifferentiation in type 2 diabetic mice

**DOI:** 10.1186/s12906-016-1208-8

**Published:** 2016-07-16

**Authors:** Yue-e Yi, Shu-yu Li, Yan-na Nie, De-xian Jia, Zhi-hui Zhang, Yan-fei Wang, Qian Wang

**Affiliations:** Beijing University Of Chinese Medicine, Beijing, 100076 China

**Keywords:** Astragalus, KKAy mice, Diabetic nephropathy, Transdifferentiation, Transforming growth factor, Alpha smooth muscle actin, E-cadherin

## Abstract

**Background:**

Astragalus injection is used by practitioners of traditional Chinese medicine to treat diabetic nephropathy (DN). The current study was conducted to determine the effect of astragalus on tubular epithelial transdifferentiation during the progression of DN in KKAy mice, as well as to investigate the molecular mechanism underlying this effect.

**Methods:**

Diabetic, 14-week-old, male KKAy mice were randomly divided into a model group and an astragalus treatment group, while age-matched male C57BL/6 J mice were selected as controls. The treatment group received daily intraperitoneal injections of astragalus (0.03 mL/10 g per day), while the model group received injections of an equal volume of saline. Mice were euthanized after 24 weeks. Serum samples were obtained from the animals in each group for blood glucose measurement. Kidney tissue samples were used for morphometric studies. The mRNA and protein expression levels of transforming growth factor beta 1 (TGF-β1), transforming growth factor beta receptor 1 (TGFβ-R1), alpha smooth muscle actin (α-SMA), and E-cadherin were evaluated using real-time polymerase chain reaction (PCR) and western blotting.

**Results:**

Astragalus significantly reduced blood glucose levels; inhibited morphological changes in the kidneys of KKAy mice; reduced mRNA and protein expression levels of TGF-β1, TGFβ-R1, and α-SMA; and increased E-cadherin expression.

**Conclusions:**

Tubular epithelial transdifferentiation plays an important role in the development of DN in diabetic mice. Administration of astragalus likely prevents or mitigates DN by suppressing tubular epithelial transdifferentiation, protecting KKAy mice from renal damage.

## Background

Diabetic nephropathy (DN) is a major microvascular complication of diabetes mellitus and the leading cause of end-stage renal disease [1]. The major pathological change associated with DN is renal interstitial fibrosis (RIF), which is characterized by renal tubular atrophy and accumulation of extracellular matrix components (ECM) in the renal glomeruli and interstitium. Transdifferentiation of tubular epithelial cells to myofibroblast-like cells has been proposed as the central link in the development of RIF [2, 3]. Transforming growth factor beta 1 (TGF-β1) is recognized as the most important cytokine in the induction of epithelial transdifferentiation [4, 5]. TGF-β1 binds to transforming growth factor beta receptor 1 (TGFβ-R1) and inhibits the expression of E-cadherin, an epithelial cell adhesion molecule, resulting in shedding of epithelial cells from the basement membrane [6, 7]. However, TGF-β1 can up-regulate the expression of alpha smooth muscle actin (α-SMA), which can stimulate the production of myofibroblast-like cells [8].

Astragalus (*Astragalus membranaceus*) has been used for centuries in traditional Chinese medicine as an immune-modulating herb. Astragalus has been widely used by clinicians as a treatment for patients with diabetes and diabetic nephropathy [9]. Polysaccharoses, astragaloside, isoflavones, and saponin glycosides are the primary constituents of astragalus extracts [10].

Recent studies have demonstrated that administration of astragalus to patients with DN can increase their glomerular filtration rate, decrease urinary protein levels, and improve renal function [11]. In addition, astragalus has an antifibrotic effect in rats, reduces the expression of TGF-β1, and inhibits ECM component synthesis [12, 13].

Therefore, the current study explored the effect of astragalus on tubular epithelial transdifferentiation through measurement of changes in protein and mRNA levels of TGF-β1, TGFβ-R1, E-cadherin, and α-SMA in the kidney tissue of diabetic mice following astragalus administration. Furthermore, the molecular mechanism underlying the efficacy of astragalus as a clinical treatment for DN should be investigated.

## Methods

### Chemicals and reagents

The astragalus for injection was purchased from the Chengdu Di’ao Jiuhong Pharmaceutical Factory (Chengdu, China).

### Experimental animals and treatment

All experiments were performed in accordance with the Guidelines on Ethical Standards for Investigations in Animals. The study was approved by the Animal Research Committee of the Beijing University of Chinese Medicine. Sixteen male KKAy mice (9–11 weeks of age) weighing 25–28 g were used in the current experiment. Eight male C57BL/6 J mice (9–11 weeks of age) weighing 23–25 g were used as age-matched controls. All mice were purchased from the Animal Center of the Chinese Academy of Medical Science (Beijing, China) and raised in the Clinical Institute of China-Japan Friendship Hospital (Beijing, China). During the experiment, the KKAy mice were allowed access to a high-fat diet (HFD) and water *ad libitum*. A control group of C57BL/6 J mice was fed a normal diet and allowed access to water *ad libitum*. At 14 weeks of age, a blood sample was obtained from the tail vein of each mouse for the purpose of blood glucose measurement. Any mouse with a blood glucose level greater than 13.9 mM was considered diabetic. The KKAy mice were randomly divided into the model group (MG, *n* = 8) and treatment group (TG, *n* = 8), which had similar distributions of average body weight and blood glucose levels. The control group consisted of C57BL/6 J mice (CG, *n* = 8). The treatment group received daily intraperitoneal injections of astragalus (0.03 mL/10 g per day), while the model group received an intraperitoneal injection of an equal volume of saline (0.03 mL/10 g per day). The mice were housed individually in plastic cages with *ad libitum* access to food and water throughout the experimental period.

Blood samples for the determination of blood glucose levels were taken from the tip of the tail every 4 weeks using BREEZE2 Blood Glucose Test Strips (Bayer HealthCare, USA). At 24 weeks of age, all mice were deprived of food pellets for 10 h and euthanized. A portion of the kidney tissue collected from each mouse was excised and frozen immediately in liquid nitrogen to prepare it for the polymerase chain reaction (PCR) and western blotting assays. The remaining portion of tissue from each mouse was fixed for hematoxylin and eosin (HE) staining, immunohistochemical staining, and observation under the electron microscope.

### Renal histological analysis

Parts of the kidney sections were fixed in 4 % buffered paraformaldehyde, embedded in paraffin, and cut into 4-μm-thick sections, which were prepared for HE staining. The remaining kidney tissue was fixed in 2.5 % buffered glutaraldehyde, postfixed with 1 % OsO4 in phosphate buffer, dehydrated by a graded series of ethanol and transferred to absolute acetone, after infiltrated in 1:1 mixture of absolute acetone and the final spurr resin mixture, transferred to 1:3 mixture of absolute acetone and the final resin mixture for 3 h and to final Spurr resin mixture for overnight, at last, specimen was placed in capsules contained embedding medium and heated at 70 °C for about 9 h. The specimen sections were stained by uranyl acetate and alkaline lead citrate for 15 min respectively and observed in transmission electron microscope (TEM).

### Immunohistochemical staining for TGF-β1, TGFβ-R1, α-SMA, and E-cadherin

Kidney sections were fixed in 4 % buffered paraformaldehyde, embedded in paraffin, cut into 4-μm-thick sections, dewaxed, washed three times with PBS for 5 min, incubated with 3 % hydrogen dioxide solution, antigen repaired with citrate buffer solution in a microwave, blocked with 3 % bovine serum albumin, and incubated with primary antibodies against TGFβ1 (1:200 dilution, Abcam, CA, USA), TGFβ-R1 (1:400 dilution, Abgent, CA, USA), α-SMA (1:500 dilution, Proteintech, CA, USA), E-cadherin (1:400 dilution, Proteintech, CA, USA) for 1 h. Next, the sections were washed three times with PBS for 5 min, after which they were incubated in goat anti-rabbit IgG bound to HRP (1:200 dilution, Zhongshan Golden Bridge, China) for 0.5 h, washed three times with PBS for 5 min, and stained with DAB for 1 min.

### Analysis of mRNA expression levels of TGFβ1, TGFβ-R1, α-SMA, and E-cadherin by real-time PCR

Total RNA was extracted from the kidney samples using Trizol (Invitrogen, CA, USA). The total RNA concentration and RNA purity were determined by measuring the OD260/OD280 ratio of each sample. RNA was reverse-transcribed using the GoScript Reverse Transcription System (Promega, USA). Primers for PCR (Table 1) were designed and synthesized by Sangon Biotech Co., Ltd. (Shanghai, China). mRNA transcripts encoding TGFβ1, TGFβ-R1, α-SMA, and E-cadherin were detected via real-time PCR using a 7500 Fast Real-Time PCR System (Thermo Fisher Scientific, Waltham, MA, USA). The PCR products were analysed using 7500 Fast System SDS software (Thermo Fisher Scientific).

**Table 1:** PCR sequences and PCR products

Name	Size	Forward Primer (5′–3′)	Reverse Primer (5′–3′)
TGF-β1	493 bp	TCCCTCAACCTCAAATTATTCA	GCGGTCCACCATTAGCAC
TGFβ-R1	172 bp	GGCGAAGGCATTACAGTGTT	TGCACATACAAATGGCCTGT
α-SMA	322 bp	GGTGCTGTCTCTCTATGCCTCTGGA	CCCATCAGGCAACTCGATACTCTTC
E-cadherin	192 bp	AGACAGGGGTGGAGGAAGTT	GGGCAGGAGTCTAGCAGAAG
β-actin	243 bp	GAAATCGTGCGTGACATTAAGG	CACGTCACACTTCATGATGGAG

### Western blot analysis for TGF-β1, TGFβ-R1, α-SMA, and E-cadherin

The lysates were clarified by centrifugation, after which supernatants were collected. Protein concentrations were determined using the bicinchoninic acid assay (BCA) method with reagents from Applygen (Beijing, China). Equivalent amounts of tissue protein (80 μg) were resolved on SDS polyacrylamide gels and transferred by electroblotting to polyvinylidene difluoride (PVDF) membranes. The membranes were blocked in 5 % (W/V) nonfat milk at room temperature for 1 h, after which they were incubated overnight at 4 °C with specific primary antibodies against TGF-β1 (1:1000 dilution, Abcam, CA, USA), TGFβ-R1 (1:1000 dilution, Abgent, CA, USA), α-SMA (1:1000 dilution, Proteintech, CA, USA), E-cadherin (1:1000 dilution, Proteintech, CA, USA), and β-actin (1:1000 dilution, Santa Cruz, CA, USA). The membranes were washed in Tris-buffered saline (TBS)-T (Tween) buffer (0.1 % TBS-T; TBS with 0.1 % Tween) and incubated with horseradish peroxidase (HRP)-linked anti-mouse secondary antibodies (1:6000 dilution). The membranes were washed in 0.1 % TBS-T, after which immunolabeled proteins were detected by enhanced chemiluminescence reagents (Applygen, Beijing, China). The density of the detected bands was analysed using Quantity One software.

### Statistical analysis

Numerical data were expressed as the mean ± standard deviation (SD) of at least three independent experiments. Differences in group means were examined using analysis of variance (ANOVA). A value of *p* < 0.05 was considered statistically significant.

## Results

### Astragalus administration controls blood glucose levels

No apparent fluctuations in behaviour or physiological appearance were noted among mice in the control group. However, mice in the model group exhibited depression, reduced activity, increased urine output, and lacklustre fur coats, all of which are typical manifestations of diabetes. The diabetes symptoms of the treatment group were milder in severity than those of the model group were.

**Fig. 1:**
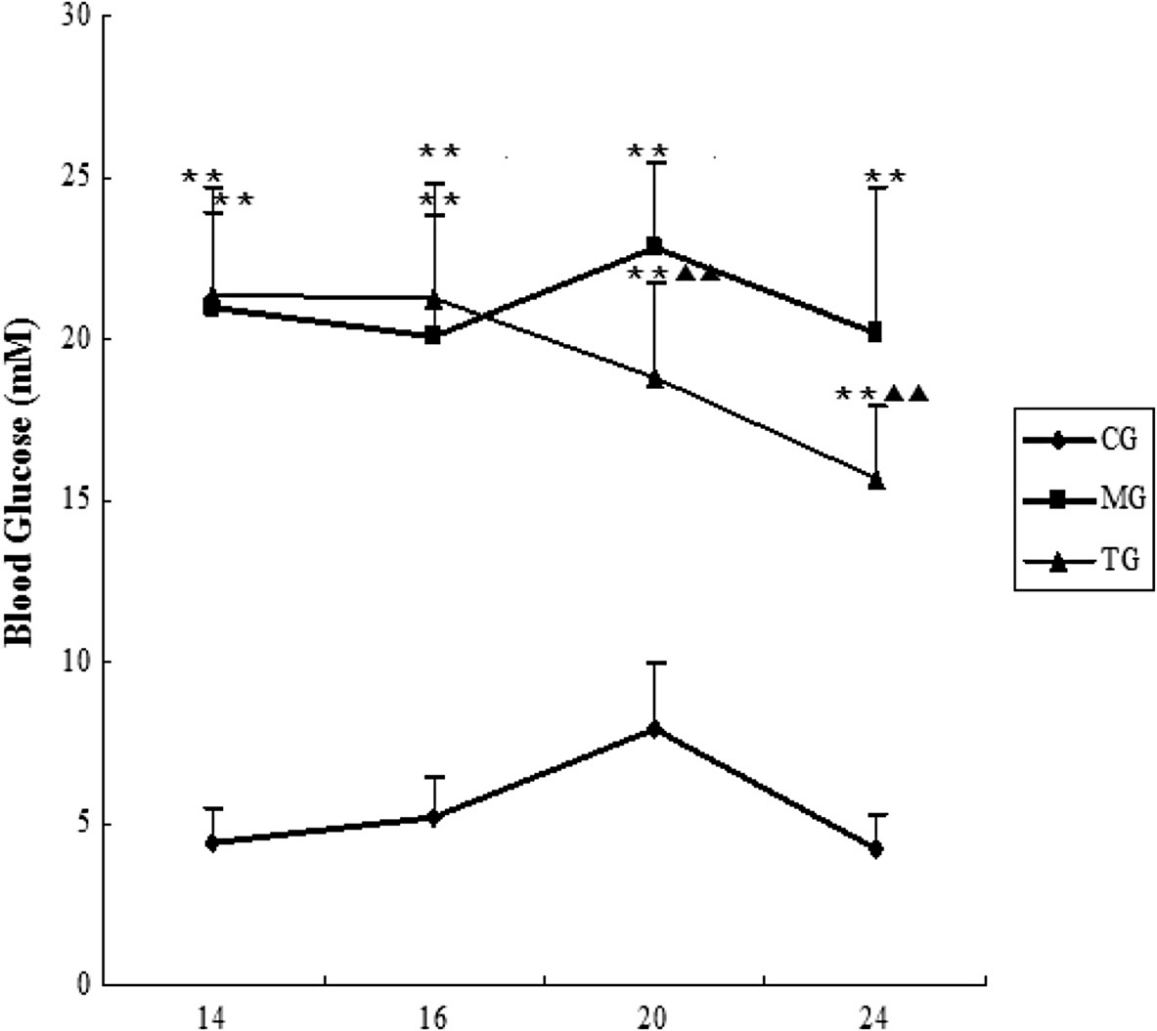
Blood glucose levels at different weeks. Data presented are means ± SD (*n* = 6–8). CG = the control group, MG = the model group, and TG = the astragalus treatment group. Compared with CG, *P < 0.05, **P < 0.01. Compared with MG, *ΔP* < 0.05, *ΔΔP* < 0.01

The blood glucose level of the model group significantly increased (*p* < 0.01) in comparison with that of the control group (Fig. 1). At 20 and 24 weeks, the blood glucose level of the astragalus treatment group significantly decreased in comparison with that of the model group (*p* < 0.01) (Fig. 1). However, astragalus treatment did not reduce the blood glucose level of the treatment group to a level within the normal range.

### Astragalus prevents morphological changes in the kidneys of diabetic mice

**Fig. 2:**
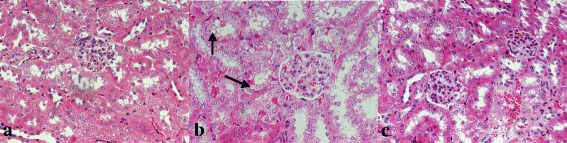
Renal pathology of the different groups. Pathological damage in KKAy mice at 24 weeks of age. 2A-2C: HE staining, 400×. The control group (**a**), the model group (**b**), and the treatment group (**c**). HE staining revealed a vacuolar degeneration in the renal tubular epithelial cells in model group

To identify pathological damage in the kidney and confirm the protective effect of astragalus in subjects with DN, kidney sections were processed for HE staining. Several DN-induced changes in renal morphology were observed in the model group, but were not present in the control group, including thickening of the basal membrane and vacuolar degeneration in renal tubular epithelial cells (Fig. 2a-c).

### Transmission electron microscopy

**Fig. 3:**
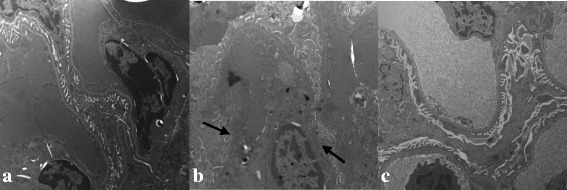
Ultrastructural change of the different groups. Ultrastructural damage in KKAy mice at 24 weeks of age. 2A-2C: 12000×. The control group (**a**), the model group (**b**), and the treatment group (**c**). Observation by TEM revealed a irregular thickening of glomerular basement membrane, effacement of foot processes in model group

TEM observation revealed that the control group showed glomerular basement membranes with defined structures and normal foot processes. However, 24-week-old model mice showed irregular thickening of the glomerular basement membrane, effacement of foot processes, and accumulation of the mesangial and renal interstitial matrix. After treatment, the severity of all of the morphological changes listed above decreased to varying degrees (Fig. 3a-c).

### Immunohistochemical staining

**Fig. 4:**
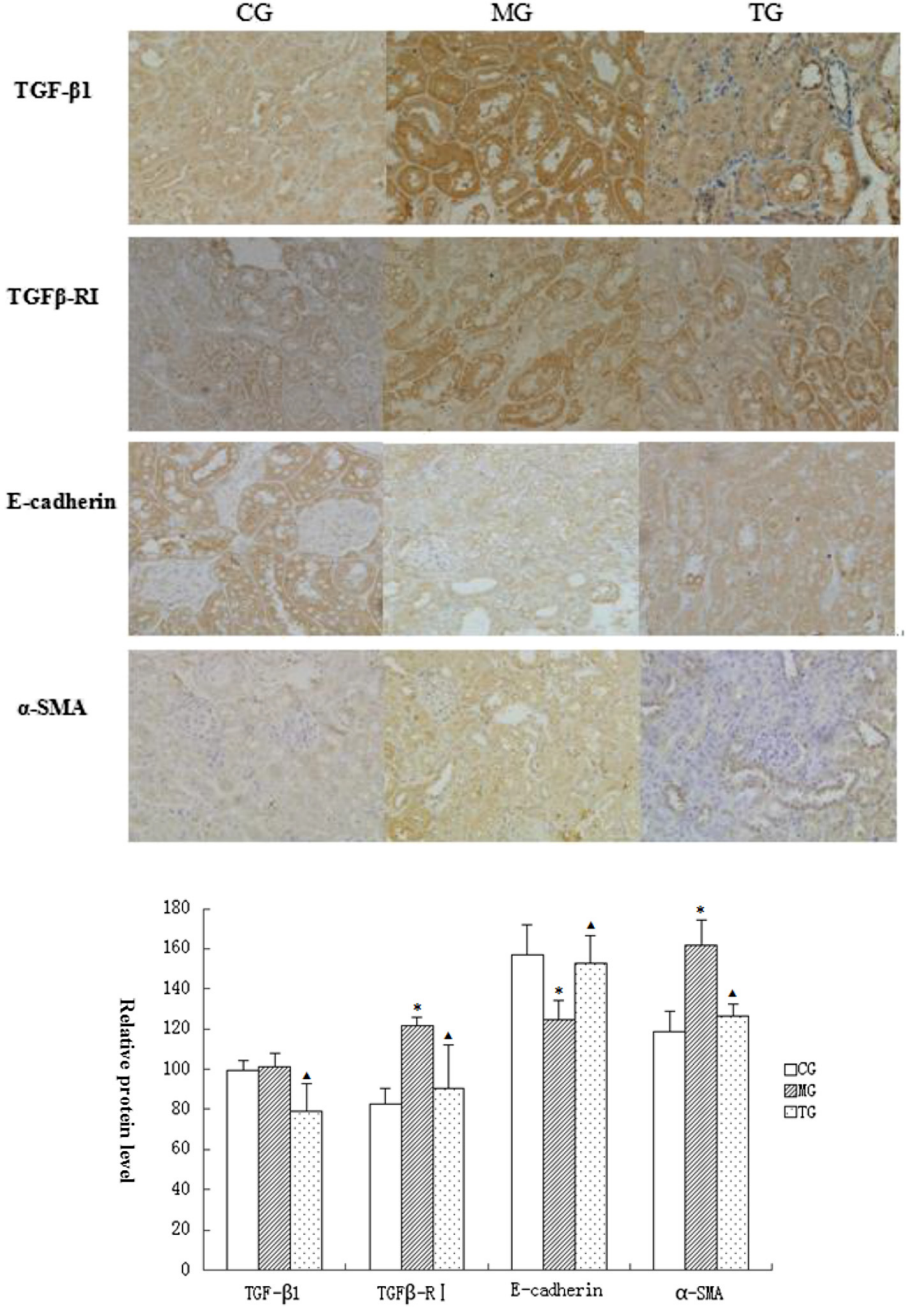
Relative protein levels of different group. analysed by Immunohistochemical staining. Data presented are means ± SD (n = 6–8). CG = the control group, MG = the model group, and TG = the astragalus treatment group. Compared with CG, *P < 0.05, **P < 0.01. Compared with MG, *ΔP* < 0.05, *ΔΔP* < 0.01

Immunohistochemical staining analysis was used to examine the expression of α-SMA, E- cadherin, TGFβ1, and TGFβ-R1. The results shows that these proteins mainly express in renal tubular epithelial cells. Analyzed by the image pro plus software,we found that the model group displayed significantly higher levels of α-SMA (*P* < 0.05), TGFβ1 (*P* < 0.05), and TGFβ-R1 (*P* < 0.05), and lower levels of E- cadherin (*P* < 0.05) when compared to the control group (Fig. 4). Treatment with astragalus significantly inhibited the expression of α-SMA (*P* < 0.05), TGFβ1 (*P* < 0.05), and TGFβ-R1 (*P* < 0.05) proteins, as well as the apparent increase in E- cadherin (*P* < 0.05) protein in diabetic mice.

### Effects of astragalus on the expression of α-SMA, E-cadherin, TGF-β1, and TGFβ-R1 at the mRNA level

**Fig. 5:**
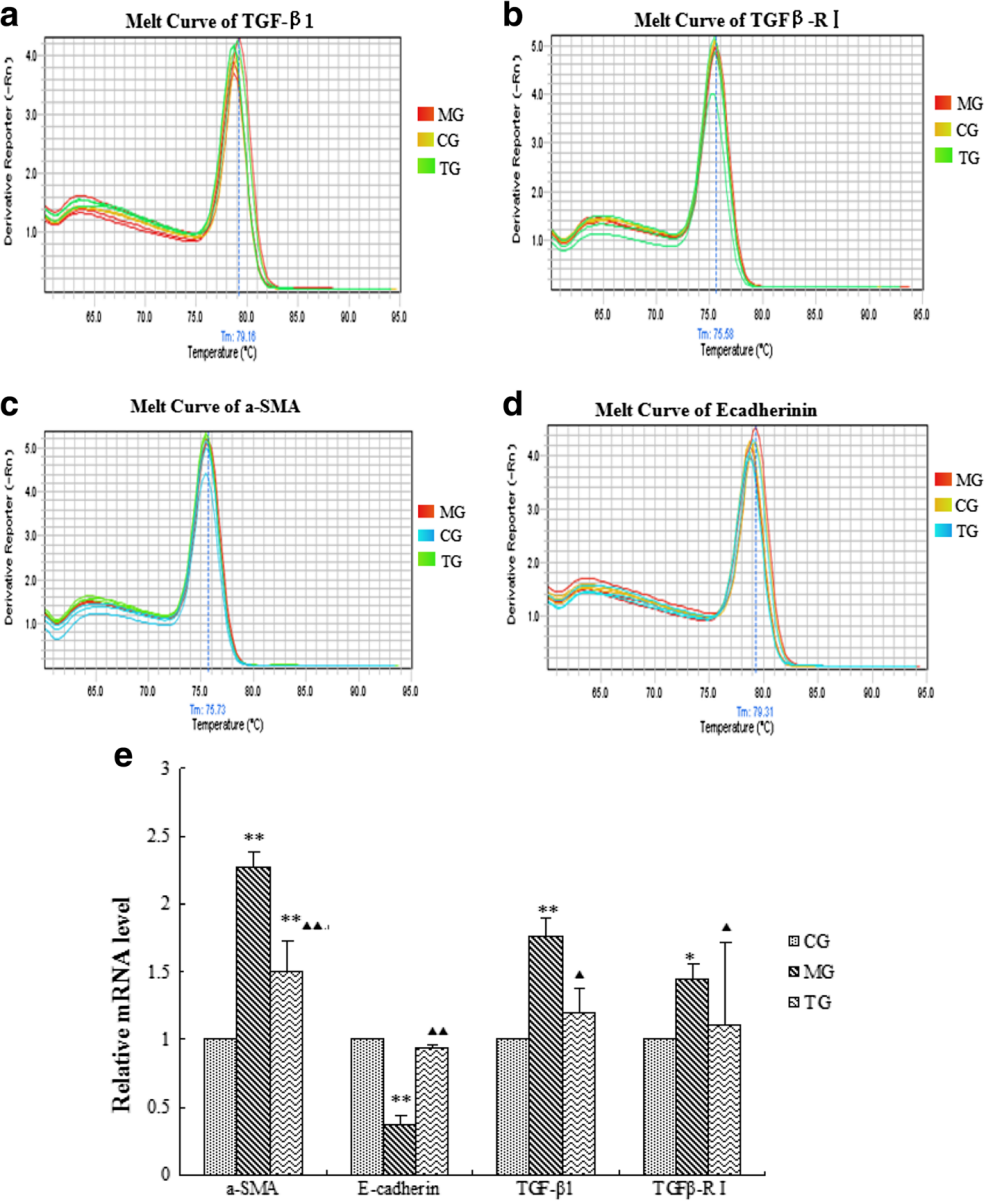
Relative mRNA levels of different group analysed by real-time PCR. **a**, **b**, **c**, **d** are respectively melt curve of TGF-β1, TGFβ-R1, α-SMA, E-cadherin. Data presented are means ± SD (n = 6 ~ 8). CG = the control group, MG = the model group, and TG = the astragalus treatment group. Compared with CG, *P < 0.05, **P < 0.01. Compared with MG, Δ*P* < 0.05, ΔΔ*P* < 0.01

Using real-time PCR, we found that administration of astragalus significantly modulated the mRNA expression levels of TGF-β1 and TGFβ-R1 in the kidneys of mice with DN. Significant reductions in the relative expression levels of TGF-β1 (*p* < 0.05) and TGFβ-R1 (*p* < 0.05) mRNA transcripts were apparent in DN mice treated with astragalus in comparison with those of the model group (Fig. 4). The relative expression level of α-SMA (*p* < 0.01) significantly decreased in DN mice treated with astragalus in comparison with that of the model group (Fig. 4). The relative expression level of E-cadherin (*p* < 0.01) significantly decreased in DN mice in comparison with that of the control group. DN mice treated with astragalus showed significantly increased E-cadherin expression (*p* < 0.05) in comparison with that of the model group (Fig. 5).

### Effects of astragalus on the expression of α-SMA, E-cadherin, TGFβ1, and TGFβ-R1 at the protein level

**Fig. 6:**
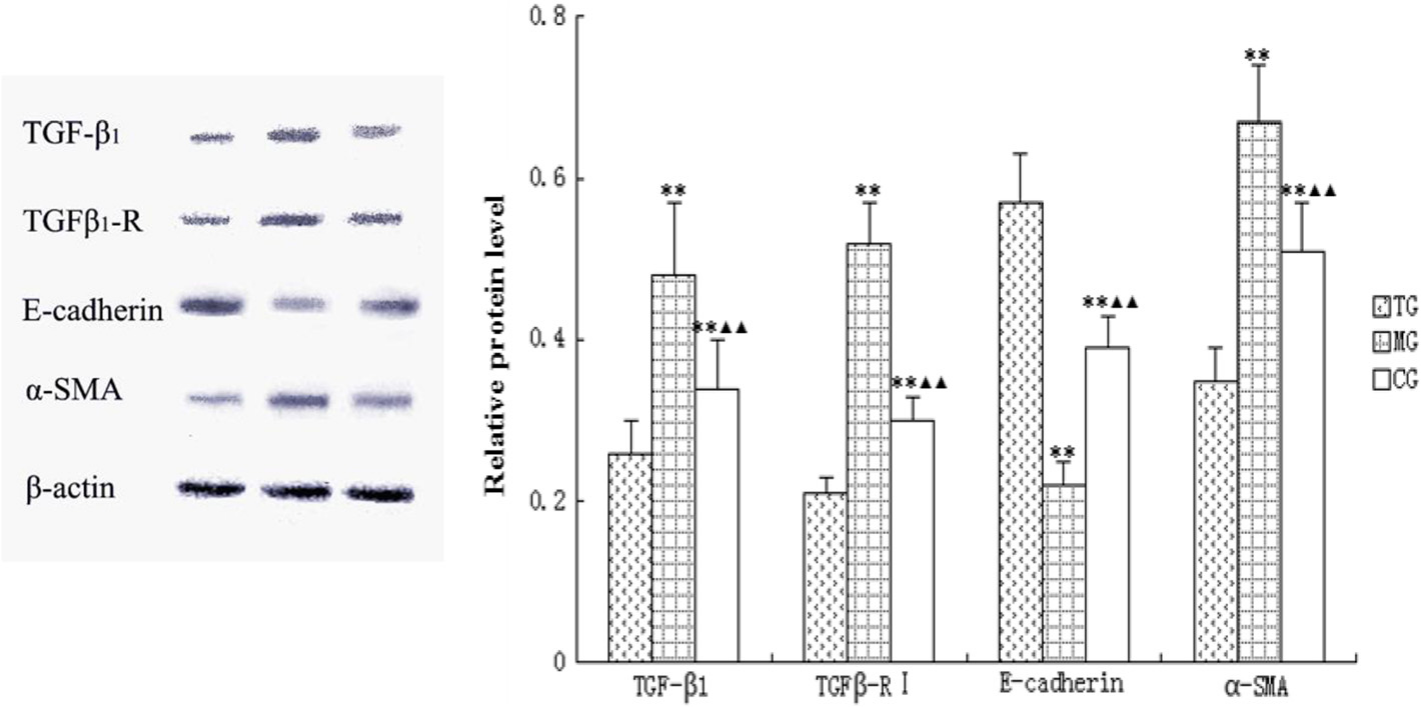
Relative protein levels of different group analysed by western-blot. Data presented are means ± SD (n = 6–8). CG = the control group, MG = the model group, and TG = the astragalus treatment group. Compared with CG, *P < 0.05, *nnP* < 0.01. Compared with MG, *ΔP* < 0.05, *ΔΔP* < 0.01

Western blot analysis was used to examine the protein expression levels of α-SMA, E-cadherin, TGF-β1, and TGFβ-R1. The model group displayed significantly higher levels of α-SMA (*p* < 0.01), TGF-β1 (*p* < 0.01), and TGFβ-R1 (*p* < 0.01), as well as a significantly lower level of E-cadherin (*p* < 0.01) in comparison with the protein expression levels of the control group (Fig. 6). In DN mice, treatment with astragalus significantly inhibited the protein expression of α-SMA (*p* < 0.01), TGF-β1 (*p* < 0.01), and TGFβ-R1 (*p* < 0.01), while it significantly (*p* < 0.01) increased E-cadherin protein expression (Fig. 6).

## Discussion

RIF is a major pathological change associated with DN and is viewed as an accurate predictor of worsening renal function. It is characterized by accumulation of ECM, degeneration of tubular epithelial cells, atrophy, thickening of basal membranes, and so on [14, 15]. For the present study, we selected the KKAy mouse, a well-established model of type 2 diabetes that was produced by transferring the yellow obese gene (Ay allele) into the KK/Ta mouse [16], KKAy mice developed obesity, hyperglycaemia, and albuminuria by 12–14 weeks of age in previous study. Moreover, HE staining demonstrated vacuolar degeneration in the renal tubular epithelial cells. Further, ultrastructural revealed irregular thickening of glomerular basement membrane, effacement of foot processes.

In the clinic, The Chinese herb astragalus is used as a treatment for patients with DN [17, 18]. The major constituents of astragalus are polysaccharoses, astragaloside, and isoflavones, have been shown to differentially lower high blood glucose and triglyceride levels, improve impaired glucose tolerance and increase insulin sensitivity in skeletal muscle in models of type 2 diabetes [19–22]. The astragalus injection used in our study formulation is a sterile aqueous solution of astragalus extract. The blood glucose levels of DN mice significantly decreased after astragalus treatment for 6 and 10 weeks. In comparison with the DN model group, the treatment group showed a milder increase in the abundance of ECM proteins. A report indicating that astragalus injections improve renal function by inhibiting tubular epithelial transdifferentiation and subsequent collagen production provides further support for these conclusions [23]. Furthermore, it was found that the treatment group exhibited milder symptoms (significantly increased urine output, slowed activity, lack of energy, and loss of hair sheen) compared to the model group following the injection of astragalus. Taken together, administration of astragalus may be appropriate for controlling blood glucose levels and reverse renal histopathology changes, which could lead to ameliorate the deterioration of renal function. Therefore, there is a necessity to explore the molecular mechanisms of astragalus administration for the treatment of DN.

We likewise aimed to investigate the mechanism of astragalus administration as a treatment for DN by focusing on the epithelial-mesenchymal transition (EMT). Transdifferentiation of tubular epithelial cells into active myofibroblasts is a central event in the pathology of RIF [2, 3]. Myofibroblasts can produce large amounts of ECM proteins, including collagen and fibronectin. The behaviour of activated myofibroblasts may determine whether fibrosis occurs in the progression of DN, but this effect is countered by the persistence of TGF-β1 signalling, which causes ECM component deposition. TGF-β1 is a key factor that initiates renal tubular epithelial cell transformation to myofibroblasts [24], it stimulates excessive synthesis and deposition of ECM proteins and participates in the mediation of phenotypic conversion of tubular epithelial cells in the pathological state, eventually leading to further atrophy and interstitial fibrosis of renal tubular epithelial cells [25, 26]. In our study, through using immunohistochemistry, we found that TGFβ1 and it’s receptor TGFβ-R1 were mainly expressed in the cytoplasm of the renal tubular epithelial cells, and were rarely expressed in glomeruli. We also discovered that the amount of TGFβ1 and TGFβ-R1 expression was higher in the model group than in the normal group, which was in accord with previously reported findings [27]. Additionally, treatment with astragalus significantly reduced TGFβ1 mRNA and TGFβ-R1 mRNA expression, which suggested that astragalus may play a role in the down-regulation of TGFβ1 and TGFβ-R1 at the transcriptional level. Our western blot analyses confirmed that TGFβ1 and TGFβ-R1 proteins were up-regulated in the model group.

In addition to TGFβ1 and it’s receptor, the E-cadherin and α-SMA also play a critical role in development of EMT. E-cadherin is a calcium-dependent transmembrane protein that mediates mutual adhesion between cells through a chain of X-linked proteins (intracellular adhesion and junction proteins) and actin filaments. It is present mainly in epithelial cells and plays an important role in maintaining kidney epithelial cell structure and polarity integrity [28, 29]. Mutual adhesion between cells decreases when E-cadherin expression is reduced or absent, resulting in dispersal of cells to the periphery. In circumstances allowing migration and invasion, dispersed cells invade other parts of the body [30]. In the process of renal tubular epithelial cell transdifferentiation, inhibition of E-cadherin expression can affect the structural integrity of tubular epithelial cells, enabling epithelial cells to be separated from adjacent cells and fall off the basement membrane. α-SMA is the characteristic protein produced by myofibroblasts after their transdifferentiation from kidney cells [31, 32]. Stationary fibroblasts do not express α-SMA, after transdifferentiation of tubular epithelial cells, the active myofibroblasts express protein markers of mesenchymal cells, including vimentin and α-SMA. Indeed, α-SMA expression in the kidney can indirectly reflect the number of myofibroblasts and the degree of RIF [33]. Therefore, expression of α-SMA provides confirmation of cellular transdifferentiation. As indicated by our results, the expression levels of E-cadherin were decreased in the kidneys of diabetic mice, and the expression of α-SMA in the kidneys of the model group was much higher than that in the kidneys of the control group or treatment group,. Furthermore, the fact that the administration of astragalus promoted the expression of E-cadherin, and suppressed the expression of α-SMA, which resulted in suppressed transdifferentiation and improved renal conditions, provides further evidence for the effectiveness of astragalus in the treatment of DN.

## Conclusions

RIF is a major pathological change in the progression of DN, the progression of RIF is a continuous, dynamic process that involves complex pathogenic and regulatory mechanisms. This study showed that intraperitoneal administration of astragalus inhibited the progression of RIF by reducing blood glucose levels; inhibiting the expression of α-SMA, TGF-β1, and TGFβ-R1; down-regulating E-cadherin expression; these results demonstrated that astragalus administration could be a potential treatment for DN, and that astragalus could improve the outcomes associated with DN by suppressing transdifferentiation.

## Abbreviations

APS, astragalus polysaccharide; ɑ-SMA, ɑ-smooth muscle actin; BCA, bicinchoninic acid assay; CG, control group; DN, diabetic nephropathy; ECM, extracellular matrix components; HE, hematoxylin and eosin; HFD, high-fat diet;MG, model group; MyoF, myofibroblasts; PCR, polymerase chain reaction; RIF, renal interstitial fibrosis; SD, standard deviation; TEM, transmission electron microscope; TG, treatment group; TGF-β1, transforming growth factor-β1; TGFβ-R1, transforming growth factorβ-Receptor 1
